# 4-Bromo-2-[(phenyl­imino)­meth­yl]phenol

**DOI:** 10.1107/S1600536814015268

**Published:** 2014-07-05

**Authors:** Xu-Xiu Yan, Li-Ping Lu, Miao-Li Zhu

**Affiliations:** a Institute of Molecular Science, Key Laboratory of Chemical Biology and Molecular, Engineering of the Education Ministry, Shanxi University, Taiyuan, Shanxi 030006, People’s Republic of China

**Keywords:** crystal structure

## Abstract

The title compound, C_13_H_10_BrNO, is essentially planar (r.m.s. deviation = 0.026 Å) and the dihedral angle between the planes of the two aryl rings is 1.5 (3)°. An intra­molecular O—H⋯N hydrogen bond generates an *S*(6) ring.

## Related literature   

For background to the biological activity of Schiff bases, see: Han *et al.* (2012 ▶); Rehman *et al.* (2008 ▶); Ritter *et al.* (2009 ▶); Vanco *et al.* (2008 ▶). For hydrogen-bond motifs, see: Bernstein *et al.* (1995 ▶).
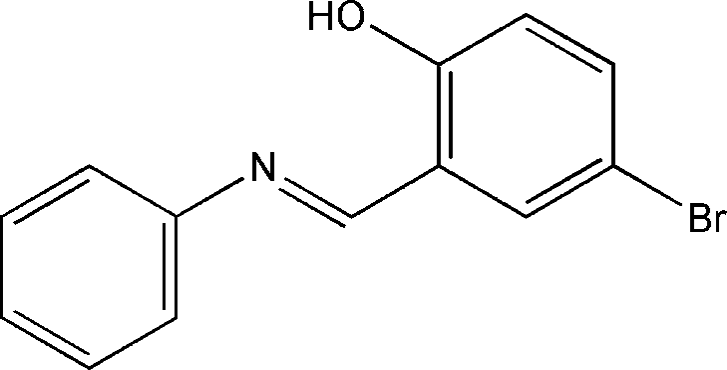


## Experimental   

### 

#### Crystal data  

C_13_H_10_BrNO
*M* *_r_* = 276.13
Orthorhombic, 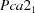

*a* = 12.353 (3) Å
*b* = 4.5092 (9) Å
*c* = 19.778 (4) Å
*V* = 1101.7 (4) Å^3^
*Z* = 4
Mo *K*α radiation
μ = 3.71 mm^−1^
*T* = 298 K
0.20 × 0.15 × 0.05 mm

#### Data collection  

Bruker SMART CCD area-detector diffractometer
Absorption correction: multi-scan (*SADABS*; Sheldrick, 2000 ▶) *T*_min_ = 0.524, *T*_max_ = 0.836
4926 measured reflections
1674 independent reflections
1444 reflections with *I* > 2σ(*I*)
*R*_int_ = 0.049

#### Refinement  

*R*[*F* ^2^ > 2σ(*F* ^2^)] = 0.039
*wR*(*F* ^2^) = 0.078
*S* = 1.04
1674 reflections
149 parameters
1 restraint
H atoms treated by a mixture of independent and constrained refinement
Δρ_max_ = 0.47 e Å^−3^
Δρ_min_ = −0.33 e Å^−3^
Absolute structure: Flack (1983 ▶), 924 Friedel pairs
Absolute structure parameter: 0.039 (18)

### 

Data collection: *SMART* (Bruker, 2000 ▶); cell refinement: *SAINT* (Bruker, 2000 ▶); data reduction: *SAINT*; program(s) used to solve structure: *SHELXS97* (Sheldrick, 2008 ▶); program(s) used to refine structure: *SHELXL97* (Sheldrick, 2008 ▶); molecular graphics: *SHELXTL/PC* (Sheldrick, 2008 ▶); software used to prepare material for publication: *publCIF* (Westrip, 2010 ▶).

## Supplementary Material

Crystal structure: contains datablock(s) I. DOI: 10.1107/S1600536814015268/nk2224sup1.cif

Structure factors: contains datablock(s) I. DOI: 10.1107/S1600536814015268/nk2224Isup2.hkl

Supporting information file. DOI: 10.1107/S1600536814015268/nk2224Isup3.cml

CCDC reference: 1010948

Additional supporting information:  crystallographic information; 3D view; checkCIF report

## Figures and Tables

**Table 1:** Hydrogen-bond geometry (Å, °)

*D*—H⋯*A*	*D*—H	H⋯*A*	*D*⋯*A*	*D*—H⋯*A*
O1—H1⋯N1	0.89 (6)	1.81 (5)	2.583 (6)	144 (5)

**Fig. 1.**
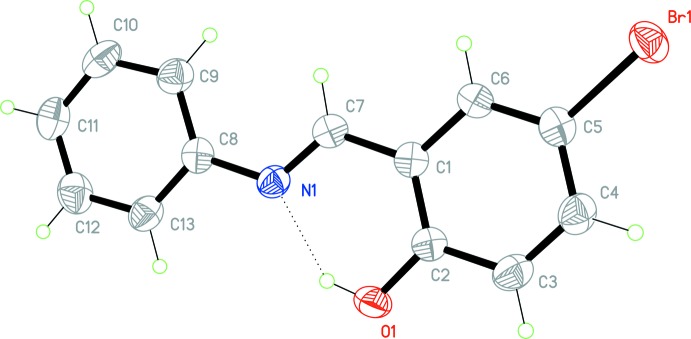
A view of the structure of (I) with displacement ellipsoids drawn at the 50% probability level. Dot line indicates hydrogen bonding interaction.

## supplementary crystallographic information

### S2.1. Synthesis and crystallization

4.0204g (20.0 mmol) 5-bromo-salicyl­aldehyde was dissolved in 30 mL of absolute ethanol. To it 1.822 mL (20.0 mmol) of aniline was added dropwise with a constant stirring. The reaction mixture was heated under refluxing for 3h. After cooling slowly, the light orange powder was separated out. The separated compound, (I), was filtered, washed thoroughly with absolute ethanol and dried in a vacuum desiccator with P_2_O_5_. Yield 91%. 0.2761g of (I) (1.0mmol) dissolved in 15 mL of absolute ethanol was heated under refluxing until thoroughly dissolved and 0.163 g (1.0 mmol) of VOSO_4_ in 5 mL of water was added dropwise with a constant stirring. The reaction mixture was adjusted to pH = 7 with NaOH solution, and then it was heated under refluxing for 3h. After cooling slowly, the yellow-green precipitates were separated out. Orange-red crystal (I) was obtained from the filtrate after two weeks. Selected IR(KBr, cm^-1^): 1614s.

### S2.2. Refinement

H atoms attached to C of (I) were placed in geometrically idealized positions with Csp^2^—H = 0.93Å. H atom attached to O of (I) was refined freely with the distance of O—H = 0.89 (6) Å.

### S3. Results and discussion

We report here the synthesis and characterization a potentially bidentate Schiff base derivative, (I), and prepared from the condensation reaction of an equimolar proportion of 5-bromo-salicyl­aldehyde and aniline in absolute ethanol. A Schiff base is condensed by primary amines and carbonyl compounds, containing strong electronegative with atoms O and N, so it is easily coordinated with metal ions to form stable complexes (Rehman *et al.*, 2008). It is reported that metal complexes of schiff base derivatives have a variety of important biological activity,such as anti-bacterial, anti-cancer, anti-tumor, hypoglycemic and so on.(Vanco *et al.*, 2008; Ritter *et al.*, 2009).Our reports indicated that copper and vanadium complexes of Schiff bases are potential inhitors over protein tyrosine phosphatases. As part of the ongoing study of vanadium complexes inhibiting protein tyrosine phosphatases (Han *et al.*, 2012), the aim of us is to synthesize new vanadium complex. Unfortunately, only the crystal structure of the title compound (I) was obtained.

The molecular structure and the crystal packing are depicted in Figure 1. X-ray structural analysis confirmed the title compound,(I), the dihedral angle between the two benzene rings is nearly 180° and and all non-H atoms are roughly coplanar with an r.m.s. deviation of 0.0255 Å for a mean plane fitted atomsin the model. There is a strong intra­molecular O—H···N hydrogen bonds with a distance of 2.583 (6) Å between donor and acceptor, which generate S(6) ring.

The strong band in IR at 1614 cm^-1^ corresponds to the C7===N1, with a bond length of 1.283 (7) Å, stretching frequency of the imine group of Schiff base.

### Crystal data

**Table:**

C_13_H_10_BrNO	*F*(000) = 552
*M**_r_* = 276.13	*D*_x_ = 1.665 Mg m^−^^3^
Orthorhombic, *P**c**a*2_1_	Mo *K*α radiation, λ = 0.71073 Å
Hall symbol: P 2c -2ac	Cell parameters from 2025 reflections
*a* = 12.353 (3) Å	θ = 2.1–26.0°
*b* = 4.5092 (9) Å	µ = 3.71 mm^−^^1^
*c* = 19.778 (4) Å	*T* = 298 K
*V* = 1101.7 (4) Å^3^	Block, orange-red
*Z* = 4	0.20 × 0.15 × 0.05 mm

### Data collection

**Table:**

Bruker SMART CCD area-detector diffractometer	1674 independent reflections
Radiation source: fine-focus sealed tube	1444 reflections with *I* > 2σ(*I*)
Graphite monochromator	*R*_int_ = 0.049
phi and ω scans	θ_max_ = 25.1°, θ_min_ = 3.3°
Absorption correction: multi-scan (*SADABS*; Sheldrick, 2000)	*h* = −13→14
*T*_min_ = 0.524, *T*_max_ = 0.836	*k* = −5→5
4926 measured reflections	*l* = −23→17

### Refinement

**Table:**

Refinement on *F*^2^	Secondary atom site location: difference Fourier map
Least-squares matrix: full	Hydrogen site location: inferred from neighbouring sites
*R*[*F*^2^ > 2σ(*F*^2^)] = 0.039	H atoms treated by a mixture of independent and constrained refinement
*wR*(*F*^2^) = 0.078	*w* = 1/[σ^2^(*F*_o_^2^) + (0.0329*P*)^2^] where *P* = (*F*_o_^2^ + 2*F*_c_^2^)/3
*S* = 1.04	(Δ/σ)_max_ < 0.001
1674 reflections	Δρ_max_ = 0.47 e Å^−^^3^
149 parameters	Δρ_min_ = −0.33 e Å^−^^3^
1 restraint	Absolute structure: Flack (1983), 924 Friedel pairs
Primary atom site location: structure-invariant direct methods	Absolute structure parameter: 0.039 (18)

### Special details

**Table:**

Geometry. All e.s.d.'s (except the e.s.d. in the dihedral angle between two l.s. planes) are estimated using the full covariance matrix. The cell e.s.d.'s are taken into account individually in the estimation of e.s.d.'s in distances, angles and torsion angles; correlations between e.s.d.'s in cell parameters are only used when they are defined by crystal symmetry. An approximate (isotropic) treatment of cell e.s.d.'s is used for estimating e.s.d.'s involving l.s. planes.
Refinement. Refinement of *F*^2^ against ALL reflections. The weighted *R*-factor *wR* and goodness of fit *S* are based on *F*^2^, conventional *R*-factors *R* are based on *F*, with *F* set to zero for negative *F*^2^. The threshold expression of *F*^2^ > σ(*F*^2^) is used only for calculating *R*-factors(gt) *etc*. and is not relevant to the choice of reflections for refinement. *R*-factors based on *F*^2^ are statistically about twice as large as those based on *F*, and *R*- factors based on ALL data will be even larger.

### Fractional atomic coordinates and isotropic or equivalent isotropic displacement parameters (Å^2^)

**Table:**

	*x*	*y*	*z*	*U*_iso_*/*U*_eq_	
Br1	0.57515 (4)	1.03271 (10)	0.49809 (4)	0.04069 (18)	
C1	0.6938 (4)	0.5393 (12)	0.3358 (3)	0.0283 (13)	
C2	0.8024 (4)	0.6143 (12)	0.3347 (3)	0.0310 (14)	
C3	0.8434 (5)	0.8206 (15)	0.3824 (3)	0.0401 (16)	
H3	0.9163	0.8720	0.3817	0.048*	
C4	0.7755 (5)	0.9449 (13)	0.4299 (3)	0.0394 (14)	
H4	0.8022	1.0819	0.4608	0.047*	
C5	0.6671 (5)	0.8650 (13)	0.4313 (3)	0.0340 (14)	
C6	0.6257 (5)	0.6708 (12)	0.3845 (3)	0.0315 (13)	
H6	0.5523	0.6255	0.3850	0.038*	
C7	0.6489 (4)	0.3280 (12)	0.2878 (3)	0.0305 (13)	
H7	0.5750	0.2881	0.2882	0.037*	
C8	0.6684 (4)	−0.0149 (12)	0.1984 (3)	0.0314 (13)	
C9	0.5609 (5)	−0.1095 (13)	0.1966 (3)	0.0379 (15)	
H9	0.5108	−0.0327	0.2271	0.045*	
C10	0.5286 (5)	−0.3203 (13)	0.1487 (3)	0.0428 (17)	
H10	0.4573	−0.3866	0.1481	0.051*	
C11	0.6026 (5)	−0.4316 (12)	0.1020 (3)	0.0401 (15)	
H11	0.5810	−0.5689	0.0695	0.048*	
C12	0.7067 (6)	−0.3361 (15)	0.1046 (4)	0.0445 (16)	
H12	0.7565	−0.4105	0.0736	0.053*	
C13	0.7409 (5)	−0.1310 (12)	0.1521 (3)	0.0373 (14)	
H13	0.8130	−0.0710	0.1528	0.045*	
N1	0.7101 (4)	0.1963 (10)	0.2449 (2)	0.0304 (12)	
O1	0.8726 (3)	0.4998 (10)	0.2894 (2)	0.0398 (10)	
H1	0.840 (4)	0.348 (13)	0.269 (3)	0.029 (17)*	

### Atomic displacement parameters (Å^2^)

**Table:**

	*U*^11^	*U*^22^	*U*^33^	*U*^12^	*U*^13^	*U*^23^
Br1	0.0408 (3)	0.0520 (3)	0.0293 (3)	0.0062 (3)	0.0020 (4)	−0.0049 (6)
C1	0.033 (3)	0.028 (3)	0.023 (3)	0.000 (3)	−0.002 (2)	0.006 (3)
C2	0.032 (3)	0.033 (3)	0.029 (4)	−0.001 (3)	0.000 (3)	0.003 (3)
C3	0.034 (4)	0.047 (4)	0.039 (4)	−0.006 (3)	−0.005 (3)	0.000 (3)
C4	0.042 (3)	0.046 (3)	0.030 (4)	−0.002 (3)	−0.007 (3)	−0.002 (3)
C5	0.040 (3)	0.034 (3)	0.028 (4)	0.002 (3)	0.000 (3)	0.004 (3)
C6	0.031 (3)	0.033 (3)	0.031 (4)	0.003 (3)	0.001 (3)	0.003 (3)
C7	0.031 (3)	0.032 (3)	0.029 (4)	−0.001 (3)	−0.002 (3)	0.005 (3)
C8	0.039 (3)	0.029 (3)	0.026 (3)	0.000 (3)	0.000 (2)	0.003 (3)
C9	0.035 (3)	0.044 (3)	0.034 (4)	−0.003 (3)	−0.005 (3)	−0.002 (3)
C10	0.039 (4)	0.038 (4)	0.051 (5)	−0.010 (3)	−0.014 (3)	0.008 (3)
C11	0.056 (4)	0.034 (3)	0.029 (4)	−0.004 (3)	−0.010 (3)	0.001 (3)
C12	0.057 (4)	0.044 (4)	0.032 (4)	0.001 (4)	0.007 (3)	−0.004 (3)
C13	0.039 (4)	0.036 (3)	0.037 (4)	−0.007 (3)	0.001 (3)	0.001 (3)
N1	0.030 (3)	0.032 (2)	0.029 (3)	−0.002 (2)	−0.002 (3)	0.000 (2)
O1	0.027 (2)	0.054 (3)	0.038 (3)	0.000 (2)	0.0047 (19)	−0.012 (2)

### Geometric parameters (Å, º)

**Table:**

Br1—C5	1.899 (6)	C8—C13	1.385 (8)
C1—C2	1.383 (7)	C8—C9	1.396 (7)
C1—C6	1.411 (8)	C8—N1	1.421 (7)
C1—C7	1.454 (8)	C9—C10	1.400 (8)
C2—O1	1.350 (7)	C9—H9	0.9300
C2—C3	1.419 (9)	C10—C11	1.394 (9)
C3—C4	1.378 (9)	C10—H10	0.9300
C3—H3	0.9300	C11—C12	1.358 (9)
C4—C5	1.387 (8)	C11—H11	0.9300
C4—H4	0.9300	C12—C13	1.384 (8)
C5—C6	1.372 (8)	C12—H12	0.9300
C6—H6	0.9300	C13—H13	0.9300
C7—N1	1.283 (7)	O1—H1	0.89 (6)
C7—H7	0.9300		
			
C2—C1—C6	119.1 (5)	C13—C8—C9	118.9 (5)
C2—C1—C7	121.4 (5)	C13—C8—N1	116.6 (5)
C6—C1—C7	119.6 (5)	C9—C8—N1	124.5 (5)
O1—C2—C1	122.6 (5)	C8—C9—C10	119.7 (6)
O1—C2—C3	117.6 (5)	C8—C9—H9	120.1
C1—C2—C3	119.8 (6)	C10—C9—H9	120.1
C4—C3—C2	120.1 (6)	C11—C10—C9	120.4 (6)
C4—C3—H3	119.9	C11—C10—H10	119.8
C2—C3—H3	119.9	C9—C10—H10	119.8
C3—C4—C5	119.8 (6)	C12—C11—C10	118.8 (6)
C3—C4—H4	120.1	C12—C11—H11	120.6
C5—C4—H4	120.1	C10—C11—H11	120.6
C6—C5—C4	120.7 (6)	C11—C12—C13	121.8 (7)
C6—C5—Br1	120.0 (4)	C11—C12—H12	119.1
C4—C5—Br1	119.2 (5)	C13—C12—H12	119.1
C5—C6—C1	120.5 (5)	C12—C13—C8	120.3 (6)
C5—C6—H6	119.8	C12—C13—H13	119.8
C1—C6—H6	119.8	C8—C13—H13	119.8
N1—C7—C1	120.6 (5)	C7—N1—C8	121.6 (5)
N1—C7—H7	119.7	C2—O1—H1	108 (4)
C1—C7—H7	119.7		
			
C6—C1—C2—O1	−179.6 (5)	C2—C1—C7—N1	2.9 (8)
C7—C1—C2—O1	0.5 (9)	C6—C1—C7—N1	−177.0 (5)
C6—C1—C2—C3	0.1 (8)	C13—C8—C9—C10	0.6 (9)
C7—C1—C2—C3	−179.8 (5)	N1—C8—C9—C10	−179.7 (5)
O1—C2—C3—C4	180.0 (5)	C8—C9—C10—C11	−1.4 (9)
C1—C2—C3—C4	0.3 (9)	C9—C10—C11—C12	1.2 (9)
C2—C3—C4—C5	0.8 (9)	C10—C11—C12—C13	−0.3 (10)
C3—C4—C5—C6	−2.2 (9)	C11—C12—C13—C8	−0.5 (10)
C3—C4—C5—Br1	178.8 (5)	C9—C8—C13—C12	0.4 (9)
C4—C5—C6—C1	2.6 (9)	N1—C8—C13—C12	−179.4 (5)
Br1—C5—C6—C1	−178.4 (4)	C1—C7—N1—C8	178.8 (5)
C2—C1—C6—C5	−1.6 (8)	C13—C8—N1—C7	176.6 (5)
C7—C1—C6—C5	178.4 (5)	C9—C8—N1—C7	−3.1 (9)

### Hydrogen-bond geometry (Å, º)

**Table:**

*D*—H···*A*	*D*—H	H···*A*	*D*···*A*	*D*—H···*A*
O1—H1···N1	0.89 (6)	1.81 (5)	2.583 (6)	144 (5)
